# Genetic diversity and population structure of *Coffea arabica* in Saudi Arabia revealed by whole-genome resequencing

**DOI:** 10.3389/fpls.2026.1942202

**Published:** 2026-09-07

**Authors:** Naser B. Almarri, Sara Osman, Mohamed M. S. Abo El-Enien, Omar A. Al-Haidar, Mohanad A. Ibrahim, Salwa M. Mostafa, Sara M. Alomran, Shakeel Ahmad, Rida Tariq, Rashed Almarri, Ibrahim Alrashidi, Nada M. Alsofuani

**Affiliations:** 1 Seed Center and Plant Genetic Resources Bank, Ministry of Environment, Water and Agriculture (MEWA), Riyadh, Saudi Arabia; 2 Food and Agriculture Organization of the United Nations (FAO), Riyadh, Saudi Arabia

**Keywords:** coffea arabica, DNA fingerprints, genetic diversity, plant genetic resources, Saudi gene bank, single nucleotide polymorphism, whole-genome resequencing

## Abstract

Gene banks are essential for conserving plant genetic resources, yet limited genomic characterization constrains their effective use in breeding. *Coffea arabica* L., an economically important crop in Saudi Arabia, remains poorly characterized at the genome-wide level. We performed whole-genome resequencing of 78 Saudi *C. arabica* accessions conserved at the Ministry of Environment, Water and Agriculture (MEWA) Seed Center and PGR Bank, generating 86 sequencing libraries, including eight technical replicates. Reads were aligned to the Cara_r6 reference genome, yielding 468, 133 variants (412, 043 SNPs and 56, 090 indels), from which 17, 452 linkage disequilibrium-pruned SNPs were retained for downstream analyses. We identified a pervasive cross-mapping artefact between the homoeologous subgenomes of this allotetraploid species, affecting 63.7% of markers and artificially inflating observed heterozygosity. Correcting this artefact reversed the inbreeding coefficient from negative (FIS ≈ −0.60) to positive (FIS = 0.37–0.52), consistent with the predominantly self-pollinating biology of *C. arabica*. Population structure analysis identified seven ancestry groups (K = 7) that were highly robust to artefact correction (Adjusted Rand Index = 0.93) but showed no correspondence with collection regions. Analysis of molecular variance attributed only 0.94% of the total genetic variation to differences among regions (p = 0.206). We developed a candidate 150-SNP fingerprinting panel with high marker informativeness (mean PIC = 0.374); however, independent validation is required before routine application because closely related accessions could not always be distinguished. These findings provide a robust genomic framework for the conservation and management of Saudi coffee genetic resources and demonstrate the importance of subgenome-aware analytical approaches for whole-genome studies of allotetraploid *C. arabica*.

## Introduction

Coffee (*Coffea* spp.) is a perennial evergreen small tree belonging to the family *Rubiaceae* and represents one of the most valuable beverage crops worldwide. Among the more than 120 Coffea species, *Coffea arabica* (2n = 4x = 44) and *Coffea canephora* (2n = 2x = 22) account for the majority of global coffee production (Omari Alzahrani et al., 2025). In 2022, global coffee production exceeded 10 million tons (green beans), with leading producers including Brazil, Vietnam, and Colombia (FAOSTAT, 2024). Originating from the highlands of East Africa, particularly Ethiopia, coffee cultivation has expanded across tropical and subtropical regions due to its high economic value, cultural significance, and adaptability to diverse agro-ecological environments (Scalabrin et al., 2020).

In the Arabian Peninsula, coffee holds profound historical and cultural importance, particularly in southwestern Saudi Arabia and Yemen, where it has been cultivated for more than four centuries in the terraced valleys of mountainous landscapes (Khemira et al., 2023b). In Saudi Arabia, ancient Arabica coffee trees, more than150 years of age, are still preserved in traditional farming systems across the regions of Jazan, Al-Baha, and Aseer, reflecting a long-standing legacy of local selection and adaptation (Alhudaib and Ismail, 2024). These historically cultivated regions are considered to harbor the greatest genetic diversity of *Coffea arabica* outside its center of origin in the Ethiopian highlands, making their germplasm an important reservoir of agronomically valuable variation. Situated along the margins of the Sahara Desert, southwestern Arabia is exposed by severe environmental conditions, including prolonged drought, high temperatures, intense solar irradiance, and desiccating winds. Continuous exposure to such stresses over centuries of cultivation suggests local accessions may possess unique adaptive traits for abiotic stress tolerance —traits that could prove increasingly valuable as climate change intensifies production challenges in traditional coffee-growing regions worldwide (Al-Ghamedi et al., 2023). Although Saudi Arabia is not a major global coffee producer, recent national initiatives promoting agricultural diversification and rural development have renewed interest in conserving and utilizing local coffee genetic resources, particularly as Saudi coffee is cultivated largely under traditional organic practices—without synthetic pesticides, herbicides, or chemical fertilizers—resulting in high-quality beans recognized among the finest specialty coffees (Tounekti et al., 2018). Indigenous accessions, largely maintained by smallholder farmers, represent a significant component of national PGR adapted to arid and semi-arid environments. Their conservation and characterization are therefore essential not only for safeguarding cultural heritage but also for strengthening agricultural sustainability and economic diversification (Almarri et al., 2026). In this context, the Seed Center and PGR Bank at the MEWA play a central role in the collection, conservation, and evaluation of Saudi coffee germplasm, laying the foundation for its effective management and future genetic improvement.

Effective conservation and breeding of coffee require a comprehensive understanding of genetic diversity, which underpins adaptability, productivity, cup quality, and resilience to biotic and abiotic stresses such as drought and heat—conditions increasingly relevant under climate change. However, germplasm collections often face challenges including misidentification, duplication, synonymy, and homonymy, which complicate conservation management and breeding efforts (Davis et al., 2025). Accurate genomic characterization is therefore essential to ensure proper curation and optimal utilization of Saudi coffee accessions.

Advances in next-generation sequencing (NGS) technologies have transformed crop genomics by enabling high-resolution characterization of genetic variation (Tariq et al., 2026). Whole genome sequencing (WGS) in particular, provides unbiased coverage of coding and non-coding regions, facilitating genome-wide discovery of single nucleotide polymorphisms (SNPs), structural variants, copy number variations, and haplotype diversity (Zhang et al., 2024). This approach is especially advantageous for species with large and complex genomes, such as allotetraploid Arabica coffee derived from interspecific hybridization, where high repetitive content and polyploid structure require comprehensive genome-wide analysis (Mekbib et al., 2022). Traditional molecular markers—including restriction fragment length polymorphisms (RFLPs) (Spaniolas et al., 2006), random amplified polymorphic DNA (RAPDs) (Nong et al., 2025), simple sequence repeats (SSRs), inter-simple sequence repeats (Andini et al., 2025), (ISSRs) (Omari Alzahrani et al., 2025), Kompetitive Allele Specific PCR (KASP) markers (Akpertey et al., 2021), and sequence-related amplified polymorphism (SRAP) markers (Al-Ghamedi et al., 2023)—have been widely used in diversity studies; however, SNPs have become the markers of choice due to their abundance, stability, codominant inheritance, and suitability for high-throughput genotyping. WGS-based SNP discovery now provides unprecedented resolution for genetic diversity analysis, genome-wide association studies (GWAS), population structure inference, pedigree analysis, and marker-assisted breeding (Zhang et al., 2023).

Despite the economic and cultural importance of coffee in Saudi Arabia, the molecular characterization of local coffee accessions remains limited. In particular, the genetic diversity and population structure of landraces and farmer-maintained accessions from southwestern Saudi Arabia have not been comprehensively explored at the whole-genome level (Khemira et al., 2023a). This knowledge gap restricts effective germplasm management, breeding advancement, and the development of climate-resilient cultivars tailored to local environments. Furthermore, SNP-based DNA fingerprinting resources for Saudi coffee germplasm are currently scarce, limiting reliable accession identification and protection of unique local varieties.

To strengthen PGR management and support sustainable coffee breeding in Saudi Arabia, this study aimed to: (i) To quantify the extent and structure of genome-wide genetic variation within the Saudi *ex situ Coffea arabica* collection and determine whether accessions maintained as distinct entries represent genetically distinguishable germplasm; (ii) characterize genome-wide genetic diversity, population structure, and variation using whole-genome resequencing; and (iii) develop high-resolution SNP-based DNA fingerprints for accurate accession identification, germplasm optimization, and future breeding applications. This study represents the first comprehensive genome-wide characterization and SNP-based fingerprinting of Saudi coffee germplasm, providing a foundational genomic resource to enhance conservation strategies and accelerate the development of improved cultivars adapted to arid environments.

## Materials and methods

### Plant material

This study utilized 78 C*. arabica* L. accessions maintained at the Seed Center and PGR Bank of the MEWA, Riyadh, Saudi Arabia. The accessions were originally collected from the major coffee-growing regions of the Kingdom, including Aseer, Al-Baha, and Jazan (**Supplementary Table 1**). Sampling was purposive and based on *ex situ* conservation availability, with all passport-verified accessions in the national collection included in the study. Eight accessions were additionally re-sequenced as independent libraries, resulting in a total of 86 sequenced libraries. These paired libraries represent technical replicates of the same physical accession (matched by Bank Code) and were used solely to validate the reproducibility of the genomic fingerprinting approach rather than as independent accessions. Because the material represents an *ex-situ* germplasm collection, the genetic clusters and population structure inferred in this study describe relationships among conserved gene-bank accessions rather than natural populations. Passport records include the region and collection site of origin but do not contain geographic coordinates or voucher specimen identifiers, as these data were not recorded during the original collection.

### DNA extraction and quality assessment

Genomic DNA was extracted from fresh young leaf tissue of 78 C*. arabica* accessions using a modified cetyltrimethylammonium bromide (CTAB) method following Doyle and Doyle (Doyle and Doyle, 1987). Approximately 1 g of leaf tissue per accession was ground into a fine powder in liquid nitrogen and incubated in preheated 2% CTAB extraction buffer (2% CTAB, 1 M Tris-HCl pH 8.0, 0.5 M EDTA pH 8.0, NaCl, and 1% β-mercaptoethanol) at 65 °C for 30 min. Following incubation, an equal volume of phenol:chloroform:isoamyl alcohol (25:24:1) was added, and samples were centrifuged at 13, 000 rpm for 10 min. The aqueous phase was recovered and further purified using chloroform:isoamyl alcohol until a clear supernatant was obtained. DNA was precipitated using sodium acetate and chilled isopropanol, followed by incubation at −20 °C for 30 min. The resulting DNA pellet was washed with absolute ethanol, air-dried, and resuspended in DNase-free water.

DNA quality and concentration were assessed using a NanoDrop 2000 Spectrophotometer (Thermo Fisher Scientific, USA), ensuring suitability for downstream library preparation.

### Library preparation, sequencing and read quality control

Sequencing libraries were prepared using the MGIEasy Universal DNA Library Preparation Kit. Briefly, 500 ng of genomic DNA per sample was fragmented to approximately 380–420 bp, quantified using the Qubit dsDNA HS Assay Kit, and verified on an Agilent 2100 Bioanalyzer. Libraries were sequenced by Shanghai Majorbio Bio-pharm Technology Co., Ltd on the DNBSEQ-T7. Raw reads were quality-filtered using fastp (v0.23.2). Across the 86 libraries, sequencing yielded a mean of 46.37 ± 2.13 million raw reads per library (range 41.80–50.65 million), of which 98.28 ± 0.28% were retained after filtering (range 97.08–98.91%), giving 13.66 ± 0.63 Gbp of clean sequence per library. Mean Q30 was 96.64% before and 97.47% after filtering. Per-sample raw and clean read statistics are given in **Supplementary Tables 2** and **S3**.

Reads were aligned to the chromosome-level reference assembly Cara_r6 of the di-haploid line ET-39 (Salojärvi et al., 2024; GenBank accession GCA_036785885.1/RefSeq GCF_036785885.1), This assembly consists of 22 pseudochromosomes representing the two subgenomes inherited from *C. canephora* (sgCC) and *C. eugenioides* (sgEE), plus unplaced contigs; organellar sequences were excluded. Chromosomes are designated chr1–chr22, where odd-numbered chromosomes belong to sgCC and even-numbered to sgEE, making chr(2k−1) and chr(2k) homoeologous pairs. The correspondence between these designations and the assembly’s native subgenome-labeled names are provided in **Supplementary Table 4**. Reads were aligned with Burrows–Wheeler Aligner (BWA-MEM, v0.7.17) using default parameters, and duplicate reads were marked with Picard MarkDuplicates (Li, 2013). Alignment quality control and per-library mapping statistics are summarized in **Supplementary Table 5** and reported in the Results.

### Variant calling and quality filtering

Variants were called from deduplicated CRAMs using GATK v4.6.1.0 HaplotypeCaller with --ploidy 2, followed by joint genotyping with GenotypeGVCFs. Variants were then filtered through a cascade of hard filters (QD < 2.0, FS > 30.0, SOR > 3.0, MQ < 40.0, MQRankSum < −3.0, ReadPosRankSum < −3.0, ALT == “*”, QUAL < 30), genotype masking to missing where per-sample depth (FMT/DP) < 2 or genotype quality (GQ) < 10, and site-level filters requiring missingness ≤ 30%, minor allele frequency ≥ 5%, mean depth between 4× and 1000×, and exactly one alternate allele (N_ALT = 1). Hardy–Weinberg tagging was applied thereafter. Summary statistics were computed using PLINK v1.9 (Purcell et al., 2007; Chang et al., 2015) and BCFtools v1.19 (Danecek et al., 2021).

The resulting dataset comprises 468, 133 variants, of which 412, 043 are SNPs and 56, 090 are indels. Only SNPs were carried forward. Sequential filtering in PLINK retained 208, 918 loci after --geno 0.05 and 207, 919 after --maf 0.05. Two linkage-disequilibrium-pruned datasets were generated: Dataset A (17, 452 SNPs, --indep-pairwise 50 5 0.5) for population structure and diversity, and Dataset B (13, 885 SNPs, --indep-pairwise 50 5 0.2) for relatedness and fingerprinting. Overall genotyping rate was 92.53% (**Supplementary Figure 1**).

### Detection and removal of subgenome cross-mapping artefact

Per-site observed heterozygosity in Dataset A displayed a striking bimodal distribution, with 3, 103 sites falling below 0.05 and 8, 106 (46%) above 0.95, while a density trough separated the modes between 0.60 and 0.70 (**Supplementary Figure 1**). Median observed heterozygosity was 0.9359. Such pervasive heterozygosity is incompatible with the predominantly self-pollinating reproductive biology of *Coffea arabica* and instead points to cross-mapping of reads between the two highly similar homoeologous subgenomes, which diverged only 350, 000–610, 000 years ago (Salojärvi et al., 2024). Four observations support this interpretation: (i) allele balance at high-heterozygosity sites is reference-skewed (median 0.409) and more overdispersed than expected under binomial sampling (sd ratio 1.16 vs. 0.88 at unaffected sites; **Supplementary Figure 2**); (ii) affected sites occur at nearly equal rates on both subgenomes (sgCC 66.18%, sgEE 61.23%), consistent with bidirectional cross-mapping; (iii) per-sample genotype missingness correlates with sequencing quality (r = –0.71 for coverage ≥4×) but shows essentially no correlation with heterozygosity (r = +0.11), indicating that the artefact is a property of sites rather than of individual libraries; and (iv) removing an equivalent number of randomly chosen sites leaves mean observed heterozygosity unchanged at 0.659 ± 0.004. We therefore filtered out all sites with observed heterozygosity > 0.70, retaining 6, 339 markers in Dataset A (A′) and 4, 868 in Dataset B (B′). Because the filter selects directly on observed heterozygosity, post-filter estimates of heterozygosity, gene diversity, PIC, and FIS are necessarily biased toward heterozygote deficit. We consequently report filtered and unfiltered values side-by-side and treat the contrast as evidence for the magnitude of the artefact rather than as an unbiased estimate. Achieving an unbiased removal would require criteria independent of genotype calls—such as mapping quality, depth, or subgenomic assignment—and remains an area for future work.

### Datasets used for each analysis

To make the basis of each result explicit, sample sizes and marker sets were tailored to each analysis. SNP discovery and variant cascade statistics were based on all 412, 043 SNPs across the 78 accessions. Nucleotide diversity (π) was estimated from unpruned SNP sets, comprising 207, 919 and 126, 199 variants for the *C. canephora* (sgCC) and *C. eugenioides* (sgEE) subgenomes, respectively, using the 75 accessions with recorded collection regions. All population-genetic summaries regional observed and expected heterozygosity (Ho, He), polymorphism information content (PIC), inbreeding coefficient (FIS), pairwise FST, and AMOVA—were computed on both the unfiltered Dataset A (17, 452 LD-pruned SNPs) and the artifact-filtered Dataset A′ (6, 339 SNPs), with N = 75 for region-based and N = 78 for accession-based analyses. Principal component analysis (PCA) and ADMIXTURE were likewise performed on Datasets A and A′ with all 78 accessions, while the maximum-likelihood phylogeny used the filtered Dataset A′ (6, 339 SNPs). Identity-by-state similarity was assessed with the filtered Dataset B′ (4, 868 SNPs). The candidate fingerprinting panel was selected from the unfiltered Dataset B (150 of 13, 885 SNPs) and is unaffected by the heterozygosity filter. Technical replicate validation employed both Dataset B′ (4, 868 SNPs) and the full Dataset B (13, 885 SNPs) across all 86 libraries (78 accessions plus 8 replicates). Finally, pathway annotation utilized all 468, 133 called variants.

### Population structure and genetic relationships

Population structure was inferred using PCA in PLINK (--pca 20) and model-based ancestry estimation in ADMIXTURE v1.3.0 for K = 2 to 10 with cross-validation (Alexander et al., 2009). Both analyses were run twice, on Dataset A and on the het-filtered Dataset A′, using identical procedures, so that the effect of the correction on inferred structure could be assessed directly.

Phylogenetic relationships were reconstructed by maximum likelihood in IQ-TREE 2 on the het-filtered alignment (78 accessions × 6, 339 SNPs), with the best-fit substitution model TVM+F+I+R2 selected by BIC and branch support from 1, 000 ultrafast bootstrap replicates.

### Genome-wide relatedness

Pairwise relatedness was assessed using Dataset B and B′. Identity-by-descent (IBD) coefficients were estimated with PLINK (--genome), and kinship coefficients were calculated using KING v2.2.7 (Manichaikul et al., 2010). Because both estimators produced degenerate estimates for this dataset, conventional relatedness thresholds were not considered appropriate. Therefore, identity-by-state (IBS) distance (PLINK DST), which does not assume Hardy–Weinberg equilibrium or rely on allele-frequency estimates, was additionally calculated and used as a relative measure of genetic similarity among accessions.

### Genetic diversity analysis

Observed and expected heterozygosity, MAF and polymorphism information content were derived from PLINK frequency and Hardy–Weinberg outputs. Nucleotide diversity, pairwise Weir–Cockerham FST and inbreeding coefficients (FIS) were computed across regional groups using VCFtools v0.1.16 (Danecek et al., 2011). Nucleotide diversity was computed on the un-pruned marker set in 50-kb windows; the value reported previously was computed on the LD-pruned set, which biases π downward. Analysis of molecular variance was performed in the R package poppr with 999 permutations, providing a significance test that the submitted analysis lacked.

PIC is reported throughout as Botstein’s statistic, PIC = 1 − (p² + q²) − 2p²q². The quantity 1 − (p² + q²) is 2pq, that is expected heterozygosity (He). These are reported as distinct quantities and are never compared to one another.

### Construction of the candidate SNP fingerprinting panel

Fingerprinting markers were selected from Dataset B by requiring a minimum call rate of 95% and a MAF ≥ 0.30, yielding 10, 300 candidate loci. A further filter of observed heterozygosity ≤ 0.60 reduced this set to 1, 116 loci. Markers were then ranked by PIC and selected on a chromosome-wise basis to generate a final panel of 150 SNPs. The selection algorithm allocated six markers to each of the 22 chromosomes (132 markers), together with six markers from single-locus scaffolds (138 markers total), while the remaining 12 markers were selected globally based on the highest PIC values. Consequently, all 12 additional markers were located on chromosome 11, resulting in a total of 18 loci on this chromosome. Because candidate loci were pre-filtered at MAF ≥ 0.30 and subsequently ranked by PIC, which is maximized as MAF approaches 0.5, the final panel is enriched for highly informative markers (mean MAF = 0.4817; maximum = 0.5000). This distribution reflects the marker selection strategy rather than the underlying allele-frequency spectrum of the germplasm collection.

The 150-SNP panel was used to generate a QR code (ISO/IEC 18004, error-correction level M) for each accession, enabling machine-readable germplasm identification. The payload format is MEWA-CA:<accession>:P150-<panel_hash>:<calls>, where the panel hash (0842B9C4) derives from the SHA-256 digest of the ordered marker list, ensuring traceability to the specific marker set, and <calls> is a 150-character genotype string (0/1/2/N) matching the marker order in **Supplementary Table 6**. All 78 codes were successfully decoded and verified byte-for-byte against the original payloads (**Supplementary Table 10**), and the full-resolution code images are provided as Supplementary Data.

### SNP annotation

Functional annotation was performed with SnpEff 5.1d against the Cara_r6 reference (Cingolani et al., 2012). Variants were screened for genes involved in caffeine biosynthesis, cell-wall carbohydrate metabolism and trigonelline biosynthesis. Because gene categories differ greatly in size and gene length, variant counts were normalised by gene span and compared using Mann–Whitney U tests with Benjamini–Hochberg correction; gene span rather than CDS length was used as the denominator, which we note as a limitation.

## Results

### Genome-wide SNP discovery and quality filtering

Variant calling identified 468, 133 variants across the 78 accessions, comprising 412, 043 SNPs and 56, 090 indels. Analyses used SNPs only. The overall genotyping rate was 92.53% and the transition/transversion ratio was 1.91 (270, 550 transitions, 141, 493 transversions; **Supplementary Figure 4**). No accession exceeded the predefined missingness thresholds of 25% or 50%. Sequential filtering retained 208, 918 loci after --geno 0.05 and 207, 919 after --maf 0.05. Linkage disequilibrium pruning generated Dataset A (17, 452 SNPs) and Dataset B (13, 885 SNPs) (**Supplementary Figure 5**).

### Identification of subgenome cross-mapping artefacts

Per-site observed heterozygosity in Dataset A exhibited a pronounced bimodal distribution, with 46% of sites showing observed heterozygosity ≥ 0.95 and a median value of 0.9359 (**Supplementary Figure 1**). A threshold of 0.70, corresponding to the trough between the two modes, was applied to identify and remove putative cross-mapping artefacts, eliminating 11, 113 of the 17, 452 markers (63.7%). These sites occurred at comparable frequencies in both subgenomes (sgCC, 66.18%; sgEE, 61.23%), while per-sample heterozygosity showed no association with sequencing quality, indicating that the elevated heterozygosity resulted from bidirectional cross-mapping between homoeologous chromosomes rather than differences in sample quality.

### SNP distribution across chromosomes and subgenomes

**Figure 1:**
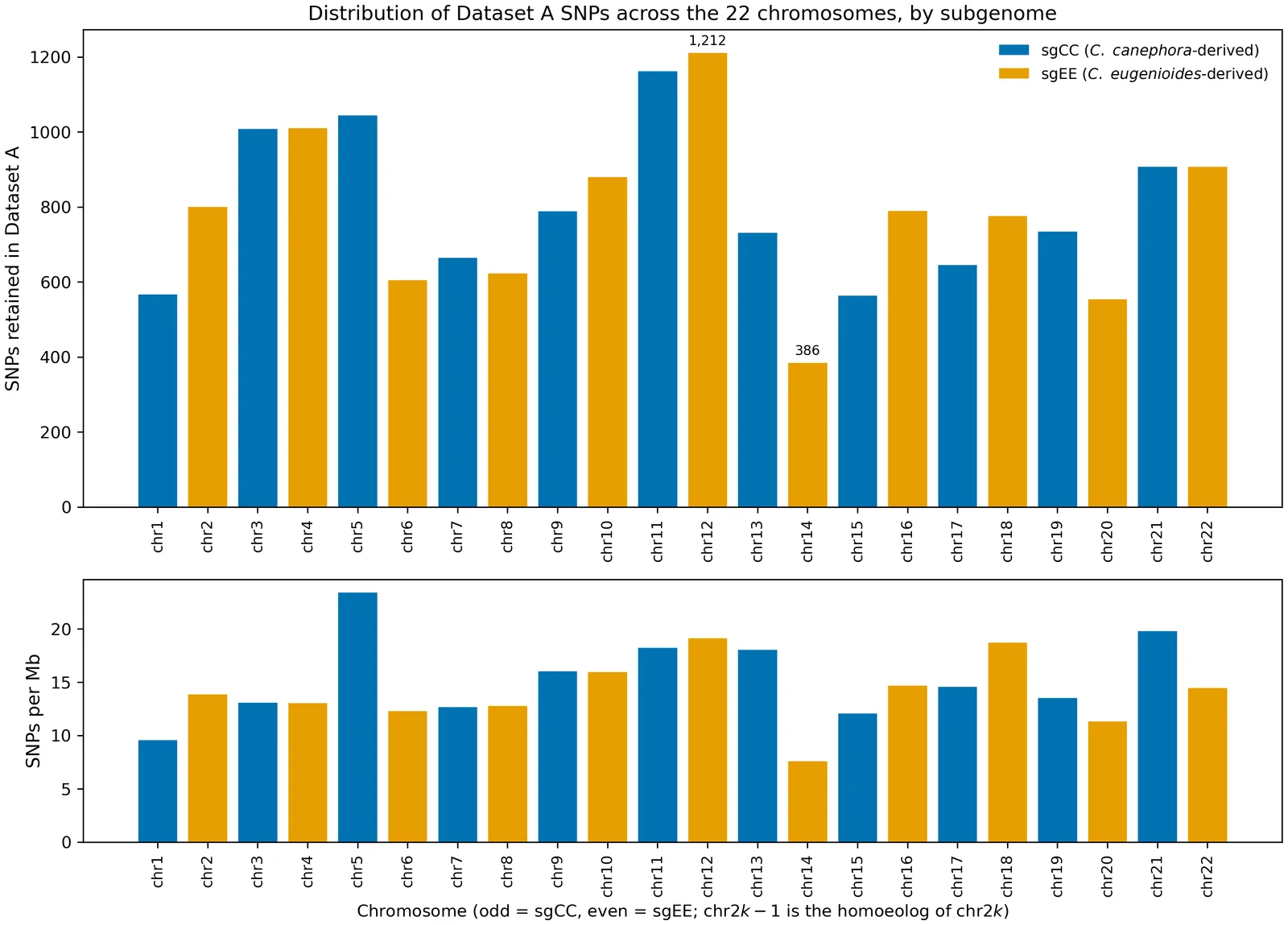
Chromosomal distribution of Dataset A SNPs across the 22 chromosomes of *Coffea arabica*. Upper panel: SNP counts; lower panel: SNP density (per Mb). Bars are coloured by subgenome (sgCC, blue; sgEE, orange). n = 78 accessions.

Dataset A markers were distributed across the 22 chromosomes and unplaced scaffolds. Among chromosome-assigned loci, chromosome 12 retained the largest number of SNPs (1, 212), followed by chromosome 11 (1, 164), whereas chromosome 14 retained the fewest (386) (**Figure 1**). Chromosomes 11 and 12 constitute a homoeologous pair (pair F), and marker density was broadly comparable between the two subgenomes, with 8, 834 chromosome-assigned markers on sgCC and 8, 559 on sgEE.

### Population structure

**Figure 2:**
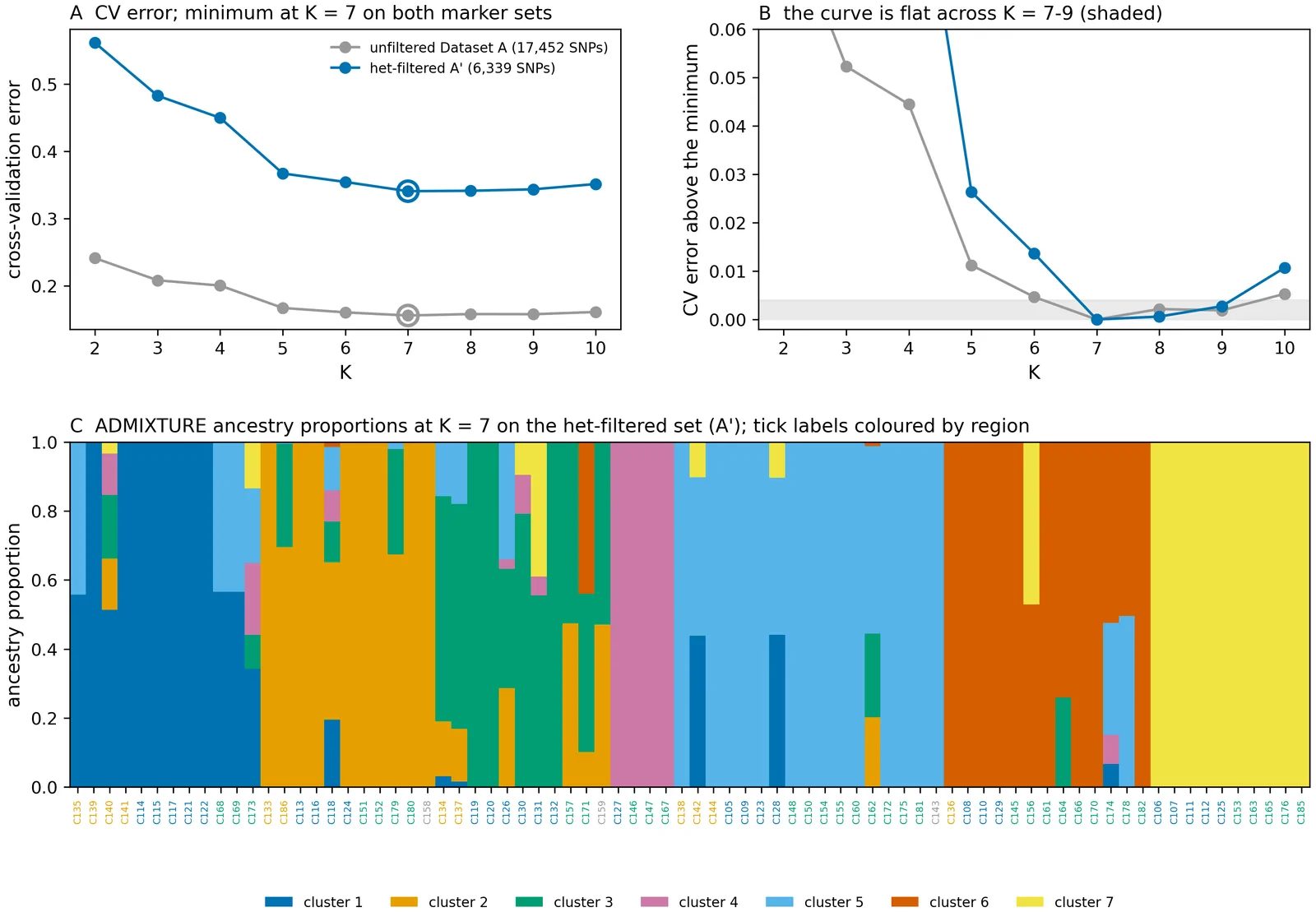
ADMIXTURE analysis of the Saudi *Coffea arabica* collection. **(A)** Cross-validation (CV) error for K = 2–10 using the unfiltered Dataset A (grey) and heterozygosity-filtered Dataset A′ (blue), with the minimum CV at K = 7 for both datasets. **(B)** CV error expressed relative to the minimum, highlighting the flat region between K = 7 and K = 9 (shaded). **(C)** ADMIXTURE ancestry proportions for the heterozygosity-filtered Dataset A′ at K = 7. Each vertical bar represents one accession, and colours indicate inferred ancestry components. Tick labels are coloured according to the region of origin. *n* = 78 accessions.

Cross-validation analysis identified K = 7 as the optimal model for both the unfiltered and heterozygosity-filtered datasets, with CV errors of 0.15609 and 0.34072, respectively (**Figure 2A**). Because the filtered dataset contained fewer markers (6, 339 vs. 17, 452 SNPs), the absolute CV values are not directly comparable. Instead, the shape of the CV curves and the position of their minima indicate that both datasets support the same optimal number of genetic clusters. In both cases, the CV curve remained relatively flat between K = 7 and K = 9 (**Figure 2B**), suggesting only marginal differences in model support across these values.

Heterozygosity filtering had minimal impact on the inferred population structure. Cluster assignments were highly consistent between the two datasets (Adjusted Rand Index = 0.9252), with 76 of the 78 accessions assigned to the same genetic cluster. Cluster sizes changed only slightly, from 19/12/12/11/10/10/4 in the unfiltered dataset to 17/13/12/11/11/10/4 after filtering, whereas the mean maximum ancestry coefficient remained similar (0.863 and 0.867, respectively). The ADMIXTURE analysis at K = 7 resolved seven genetic clusters, although several accessions exhibited admixed ancestry, indicating shared ancestry among conserved accessions rather than complete genetic separation (**Figure 2C**).

Notably, the seven genetic clusters did not correspond to the three collection regions, as each cluster included accessions from Aseer, Al-Baha, and Jazan (**Figure 2C**). This pattern is consistent with the low regional differentiation observed in the FST and AMOVA analyses and indicates that the inferred structure reflects genetic relationships among the *ex-situ* accessions, likely resulting from shared ancestry, clonal propagation, and germplasm exchange among regions, rather than geographic subdivision.

### Phylogenetic relationships

**Figure 3:**
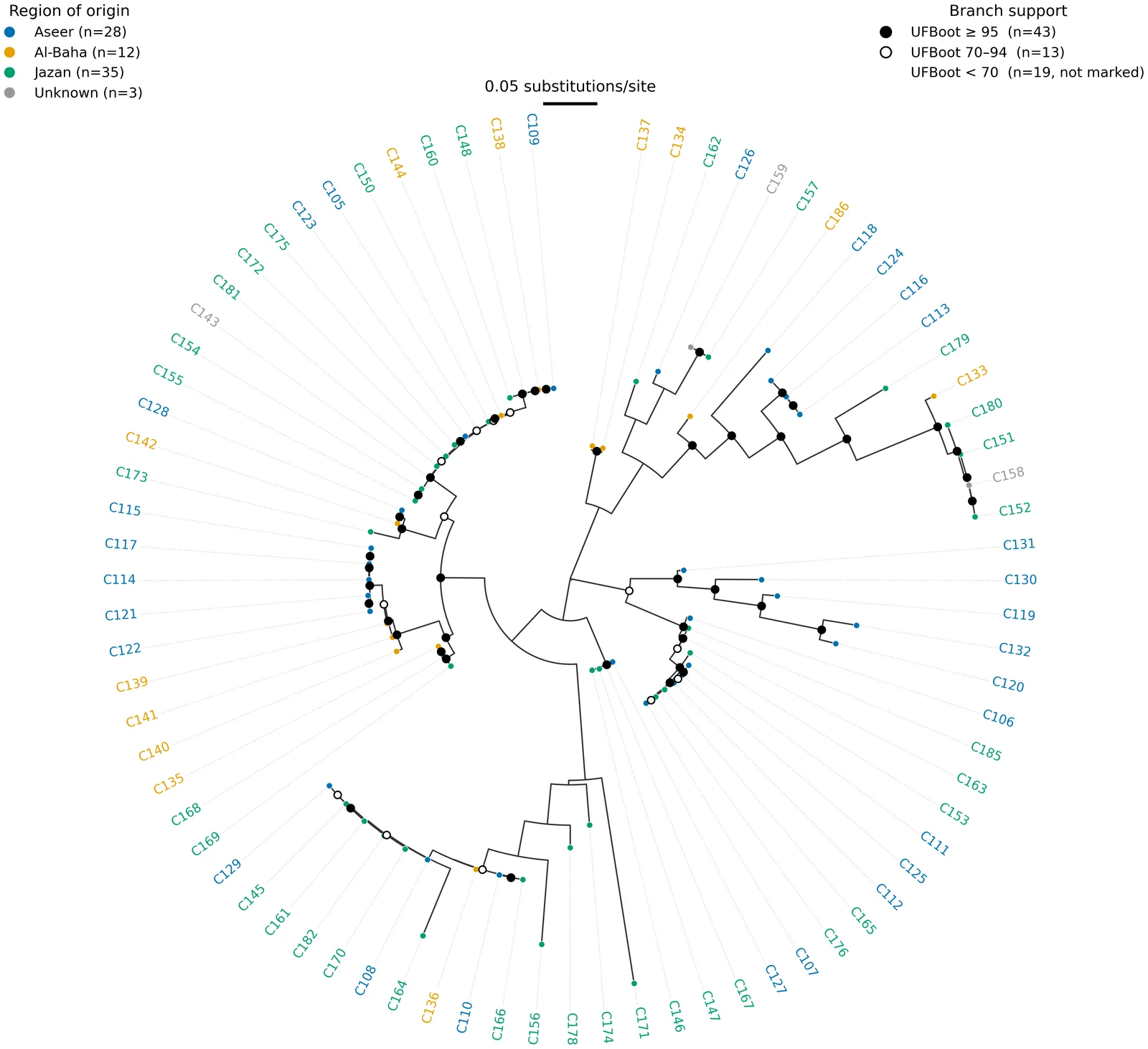
Maximum-likelihood phylogeny of 78 *Coffea arabica* accessions inferred from 6,339 heterozygosity-filtered SNPs. Tip labels and symbols are coloured according to the original collection region (Aseer, Al-Baha, and Jazan). Filled circles indicate internal nodes with ultrafast bootstrap support ≥95%, open circles indicate support between 70% and 94%, and unmarked nodes have support <70%. The tree is displayed using midpoint rooting for visualization only, as no outgroup was included.

A maximum-likelihood phylogeny reconstructed from the heterozygosity-filtered dataset (78 accessions × 6, 339 SNPs) showed moderate overall branch support (**Figure 3**). Of the 75 internal nodes, 43 (57%) received ultrafast bootstrap support ≥95%, 13 (17%) had support between 70% and 94%, and 19 (25%) had support <70%. Bootstrap support was generally high for terminal branches but lower for deeper nodes, indicating that relationships among closely related accessions were well resolved, whereas deeper evolutionary relationships remained uncertain. Accessions from Al-Baha, Aseer, and Jazan were interspersed throughout the tree, with no evidence of clustering by geographic origin. Comparison with the phylogeny inferred from the unfiltered dataset showed a bipartition concordance of 0.689, indicating that removal of putative cross-mapping artefacts influenced tree topology. Because no outgroup was available, the phylogeny is shown as a midpoint-rooted tree for visualization, whereas the corresponding equal-angle unrooted tree is presented in **Supplementary Figure 6**.

### Principal component analysis

**Figure 4:**
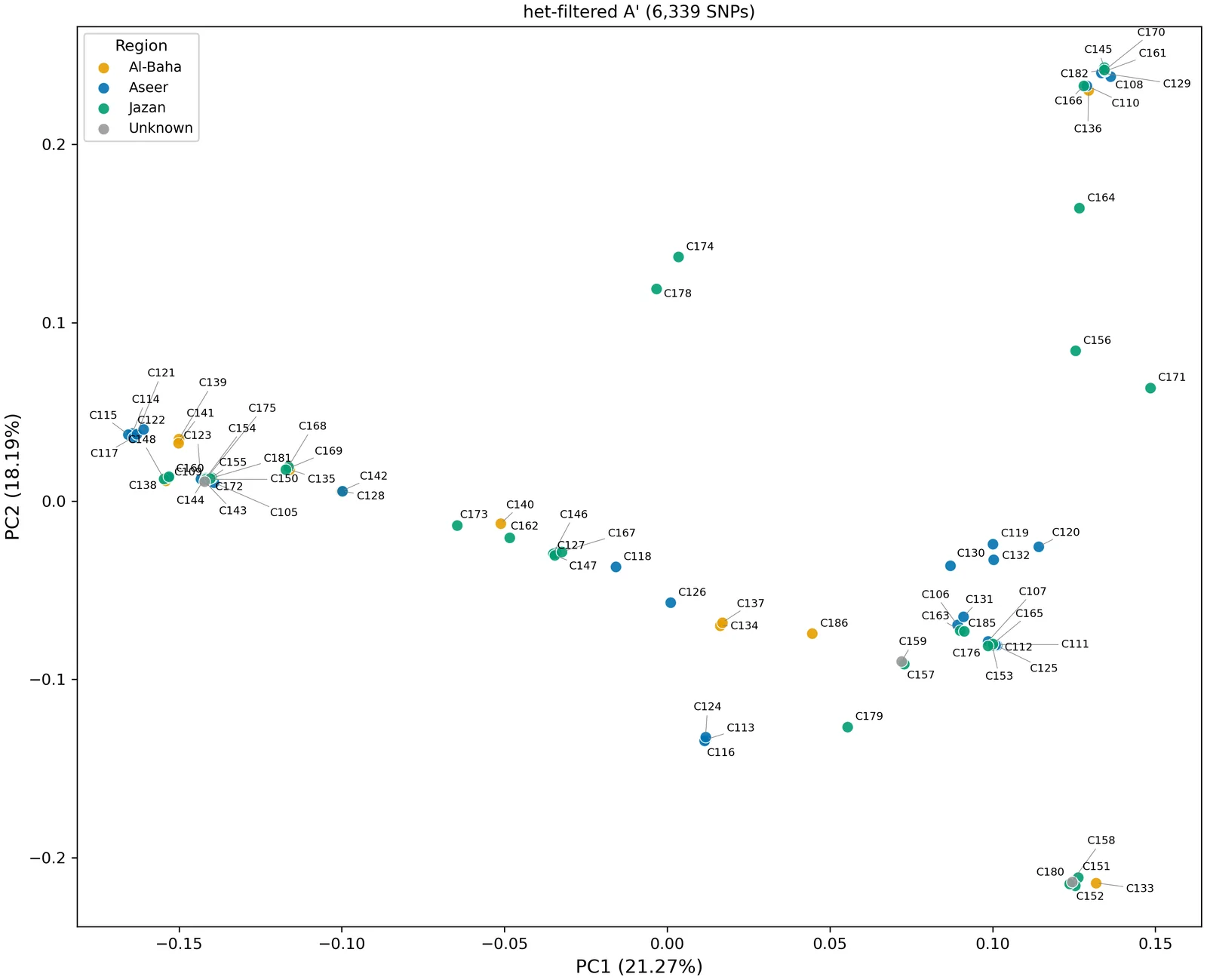
Principal component analysis (PCA) of 78 *Coffea arabica* accessions based on 6,339 heterozygosity-filtered SNPs. PC1 and PC2 explain 21.27% and 18.19% of the variation captured by the first 20 principal components, respectively. Points are coloured according to the original collection region; accessions with unrecorded origin are shown as Unknown. Labels are offset where necessary to improve readability.

PCA of the heterozygosity-filtered dataset showed that PC1 and PC2 explained 21.27% and 18.19% of the total variation captured by the first 20 principal components, respectively (**Figure 4**). The corresponding values for the unfiltered dataset were similar (20.76% and 18.01%, respectively; **Supplementary Figure 7**), indicating that heterozygosity filtering had little effect on the overall pattern of genetic variation. Accessions from Aseer, Al-Baha, and Jazan overlapped extensively in the PCA space, with no distinct clustering according to the original collection region. This pattern agrees with the ADMIXTURE analysis and supports the low level of regional genetic differentiation observed in the FST and AMOVA analyses.

### Genome-wide relatedness

Both IBD- and KING-based relatedness estimators produced degenerate results before and after heterozygosity filtering. Before filtering, 86.58% of pairwise comparisons had Z1 = 0, whereas after filtering 90.18% had PI_HAT = 0 and Z0 = 1, indicating that the estimators converged to boundary solutions rather than providing informative measures of relatedness. Similarly, 67.6% of KING kinship estimates were < −1 after filtering, falling outside the interpretable range. These patterns were consistent across heterozygosity thresholds of 0.60, 0.70, and 0.80, indicating that adjustment of the filtering threshold did not resolve the problem (**Supplementary Figure 8**).

**Figure 5:**
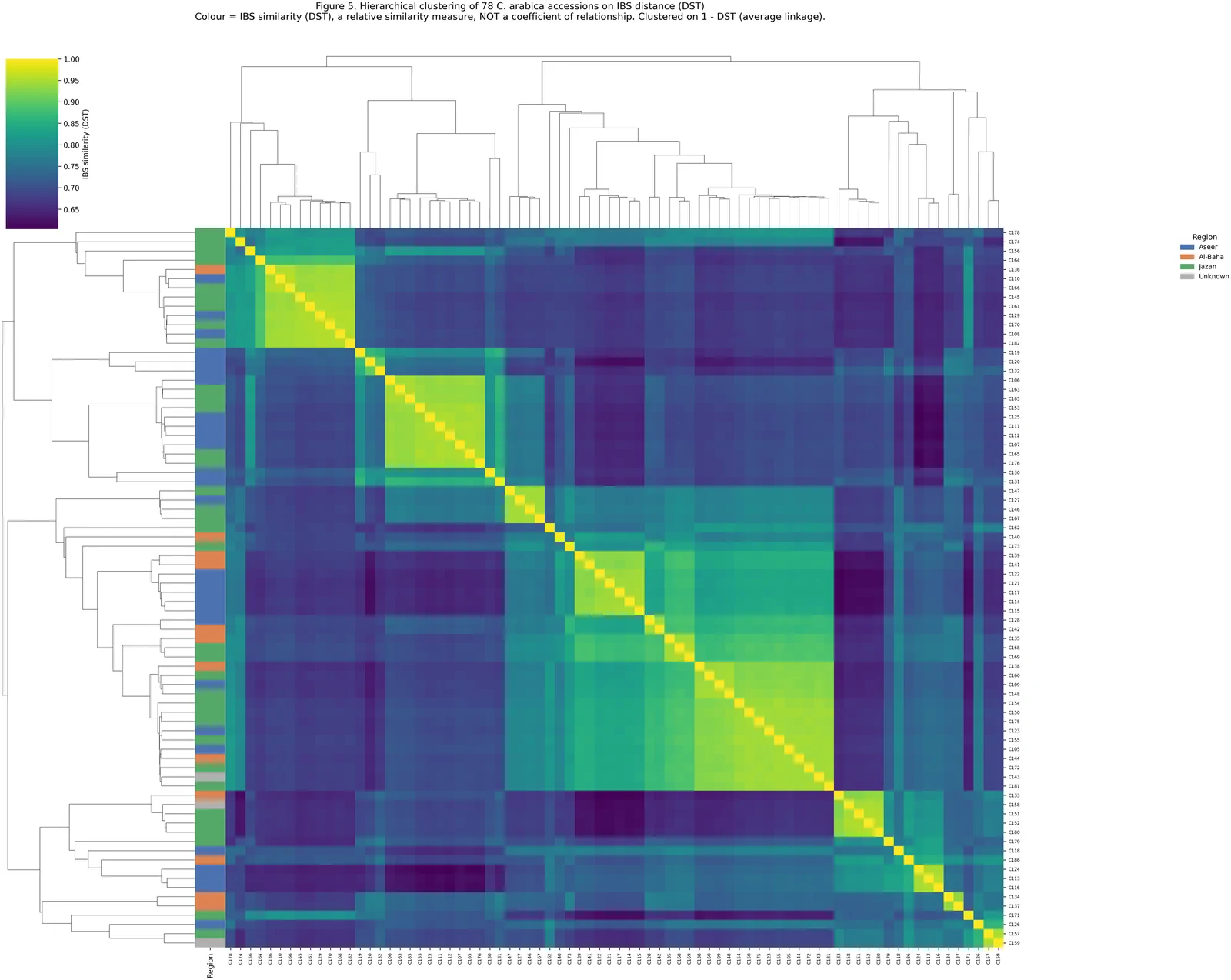
Hierarchically clustered identity-by-state (IBS) similarity matrix of 78 Coffea arabica accessions based on 4,868 heterozygosity-filtered SNPs. Technical replicate pairs (n = 8) were excluded. IBS similarity is the proportion of alleles shared between two accessions (PLINK DST); higher values indicate greater similarity. It is shown as a relative measure of genetic similarity, and the diagonal was set to the maximum observed value for visualization.

We therefore used identity-by-state (IBS) similarity—the proportion of alleles shared between two accessions as a relative measure of genetic resemblance—because it does not rely on Hardy-Weinberg equilibrium or allele-frequency assumptions. Although PLINK labels this quantity DST, it is a similarity measure (higher values indicate greater allele sharing), and we have revised our terminology accordingly. IBS similarity values were well distributed (range 0.604–0.958; median = 0.706) and highly consistent before and after heterozygosity filtering (Spearman’s ρ = 0.97; **Supplementary Figure 9A**). The similarity matrix revealed several closely related clusters of accessions embedded within a broadly continuous background of genetic resemblance, with each cluster containing accessions from Aseer, Al-Baha, and Jazan (**Figure 5**). The highest DST value for a non-replicate pair (C110–C166, 0.9583) marginally exceeded that of the closest technical replicate pair (0.9580). High DST values should therefore be interpreted as evidence of close genetic similarity, not clonal identity.

### Regional diversity and genetic differentiation

**Figure 6:**
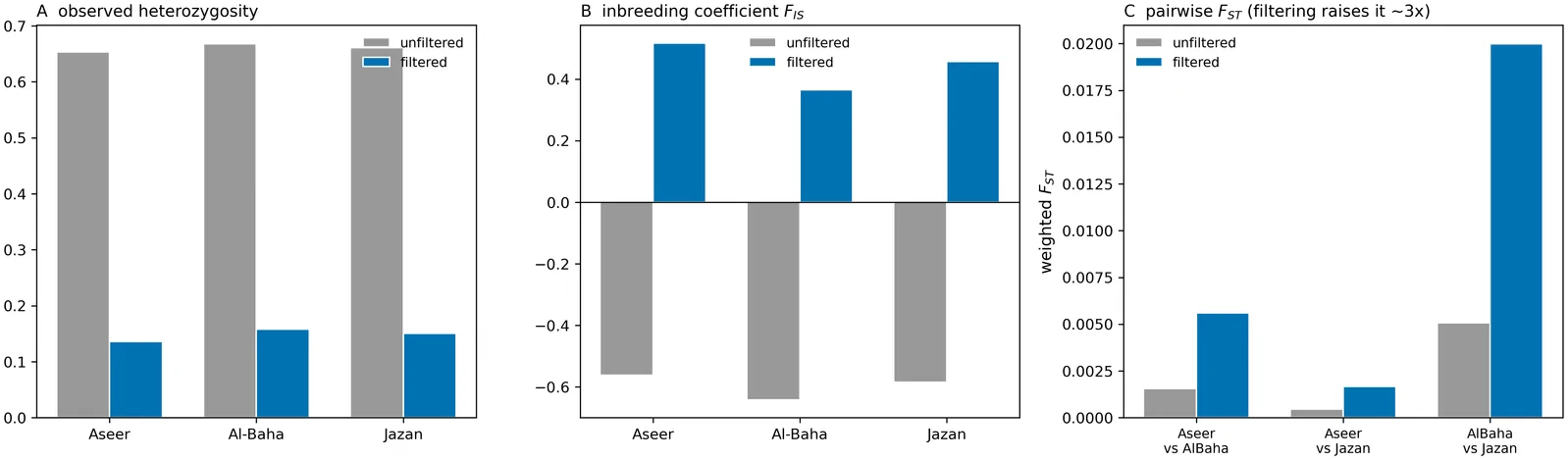
Regional genetic diversity and differentiation before and after removal of putative cross-mapping artefacts. **(A)** Observed heterozygosity (Ho). **(B)** Inbreeding coefficient (FIS). **(C)** Pairwise weighted FST among the Aseer, Al-Baha, and Jazan collections. Analyses were based on the 75 accessions with recorded collection regions.

Regional analyses were performed on the 75 accessions with recorded collection regions (Aseer, *n* = 28; Al-Baha, *n* = 12; Jazan, *n* = 35). Diversity statistics are presented before and after removal of putative cross-mapping artefacts because the effect of filtering constitutes a biological result (**Figures 6A, B**).

Removal of high-heterozygosity sites reversed the inbreeding coefficient (FIS) from an apparent heterozygote excess to a heterozygote deficit, consistent with the predominantly self-pollinating reproductive system of *C. arabica*. Random removal of an equivalent number of markers had little effect on observed heterozygosity (Ho = 0.659 ± 0.004), indicating that the observed shift resulted from elimination of cross-mapping artefacts rather than from reduced marker number alone.

Mean nucleotide diversity (π), estimated from the unpruned SNP dataset, ranged from 1.06 × 10^−4^ to 1.09 × 10^−4^ before filtering and from 5.7 × 10^−5^ to 6.3 × 10^−5^ after filtering. These estimates remain substantially higher than our previous value (~1.3 × 10^−5^), which was calculated from an LD-pruned dataset and therefore underestimated genome-wide diversity.

Regional genetic differentiation was consistently low. Pairwise weighted FST values ranged from 0.0005 to 0.0051 before filtering and from 0.0017 to 0.0200 after filtering, with the greatest differentiation observed between the Al-Baha and Jazan collections in both datasets (**Figure 6C**). Removal of putative cross-mapping artefacts increased FST estimates by eliminating sites that were heterozygous across nearly all accessions and therefore contributed little to between-region differentiation. Despite this increase, regional differentiation remained low overall.

Analysis of molecular variance (AMOVA) attributed 0.856% of the total genetic variation to differences among regions before removal of putative cross-mapping artefacts (Φ = 0.00856) and 0.944% after filtering (Φ = 0.00944). In both analyses, the among-region component was not statistically significant (999 permutations, *p* = 0.206; *n* = 75). Although pairwise FST estimates increased following artefact removal, the proportion of variance explained among regions remained largely unchanged, indicating that genetic differentiation among the three regional collections is minimal (**Table 1**).

**Table 1:** Analysis of molecular variance (AMOVA) among and within Saudi coffee accessions.

Dataset	Source of variation	d.f.	Sum of squares	Variance component	% variation	Phi stat	p-value
Dataset A	Between samples	2	7466.332	26.864	0.856	0.00856	0.206
Dataset A	Within samples	72	224017.646	3111.356	99.144		
Dataset A	Total	74	231483.979	3138.22	100		
Dataset A’	Between samples	2	6485.23	25.31	0.944	0.00944	0.206
Dataset A’	Within samples	72	191288.295	2656.782	99.056		
Dataset A’	Total	74	197773.525	2682.091	100		

df, degrees of freedom; Phi_stat, Phi-statistic.

### A candidate SNP fingerprinting panel

**Figure 7:**
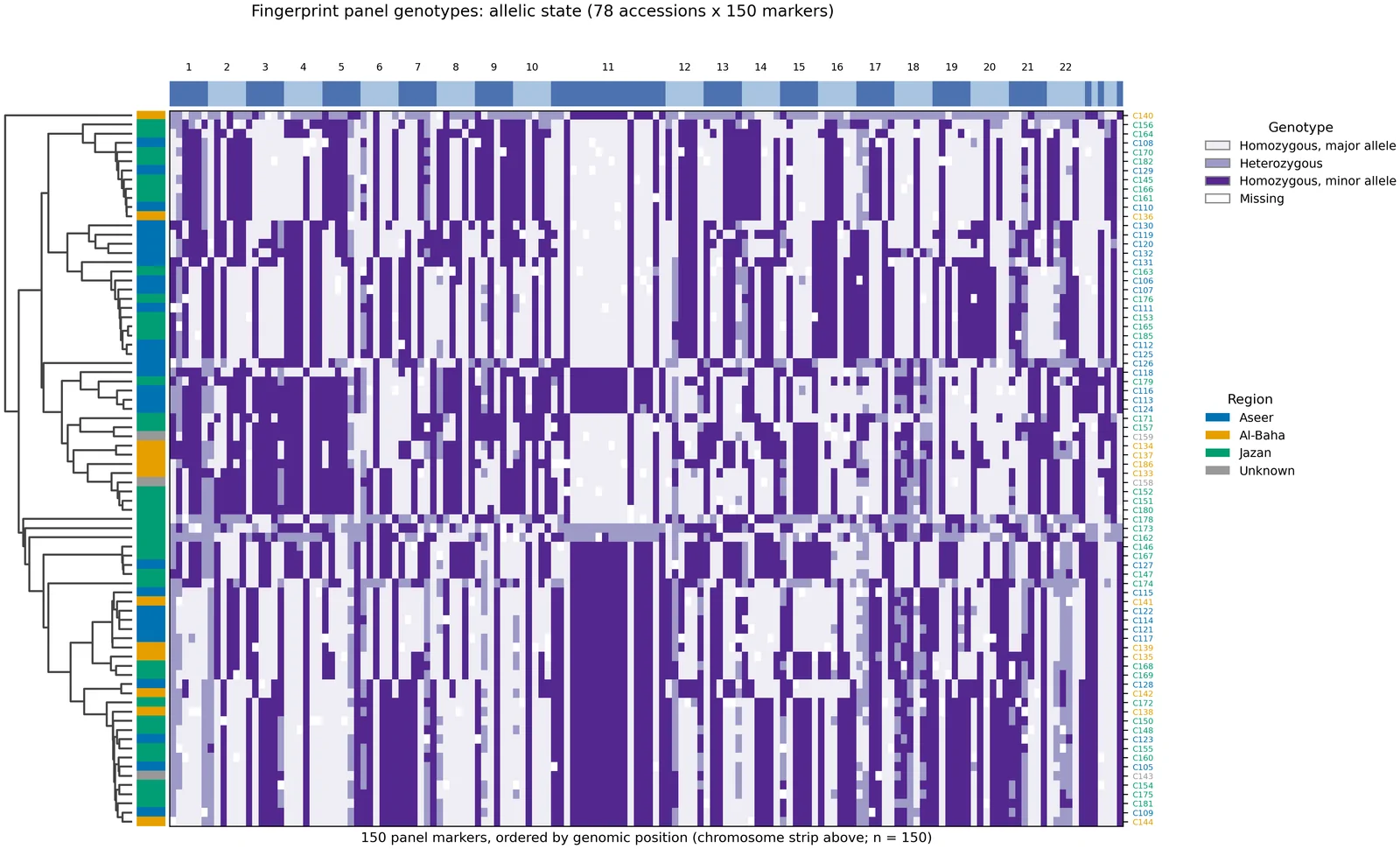
Genotype matrix of the 150-SNP candidate fingerprinting panel across 78 *Coffea arabica* accessions. Colours indicate genotype state at each marker (major homozygote, heterozygote, minor homozygote, or missing genotype). Accessions (rows) are hierarchically clustered using Hamming distance, whereas markers (columns) are ordered by genomic position following linkage disequilibrium pruning (r² ≤ 0.2). The coloured strip above the matrix indicates chromosome identity, and the left-hand annotation and accession labels indicate the original collection region. Heterozygous genotypes comprise 9.4% of the matrix, consistent with the panel mean observed heterozygosity of 0.096.

A panel of 150 SNPs was selected from Dataset B (**Figure 7**) (**Supplementary Tables 6** and **S7**). It exhibited a mean minor allele frequency (MAF) of 0.4817 (range 0.3067–0.5000), mean expected heterozygosity of 0.4974, and mean polymorphism information content (PIC) of 0.3736, reaching 99.6% of the theoretical maximum for biallelic markers (0.375). Mean observed heterozygosity was low (0.096), reflecting the deliberate selection of markers with reduced cross-mapping artefact effects; all 150 SNPs passed the artefact filter.

Technical replicate pairs were defined *a priori* from gene bank records (by shared bank code, not from genotype data); each replicate library is linked to its parent accession in **Supplementary Table 9**. All 3, 655 pairwise comparisons among the 86 libraries were ranked by identity-by-state (IBS) similarity. The eight declared replicate pairs occupied ranks 2, 13, 31, 57, 73, 75, 156, and 401; they were among the most similar pairs but did not exclusively occupy the top ranks, and the single highest similarity overall belonged to a non-replicate pair (C110–C166, 0.9583 vs. the best true replicate at 0.9580). Because the distributions of replicate and non-replicate IBS values overlapped across their entire range (**Supplementary Figure 9**), no similarity threshold can cleanly separate them; we therefore refrain from proposing a discrimination cut-off that the data do not support. Replicate concordance ranged from 71.43% to 96.38%, and results were consistent on both filtered and unfiltered marker sets, confirming that panel performance was unaffected by the cross-mapping artefact.

One replicate pair, C142–C62 (Bank Code 3157), achieved only 71.43% concordance and ranked 401st among all pairs, well below the other seven replicates. The discrepancy cannot be attributed to technical factors we examined: the pattern persists on the unfiltered marker set, and genotype missingness, per-sample heterozygosity, and sequencing, mapping, and coverage statistics for both libraries are unremarkable (**Supplementary Tables 2**, **S3**, and **S5**). Notably, C142’s closest partner is C128 (IBS similarity 0.9447, panel concordance 95.24%, with the next-best partner at 74.48%), whereas C62 has no comparably close match. Since C142 and C128 carry different bank codes and originate from different regions, a sample swap or mislabeling during library preparation is the most plausible remaining explanation. This case therefore constitutes an unresolved limitation of the candidate panel: with one of eight replicate pairs unexplained, the panel’s demonstrated reproducibility currently rests on seven pairs, and independent validation on an unrelated accession set is required before deployment.

The empirical distribution of pairwise concordance further revealed substantial genetic redundancy within the collection. Among 3, 647 non-replicate comparisons, mean concordance was 44.84%, yet one non-replicate pair (C165 and C185) was identical across all 146 successfully genotyped loci. Both accessions originated from Jazan, shared the local name Khulani, and were collected from different sites, consistent with clonal propagation of the same landrace or duplicate germplasm entries. Overall, 66 non-replicate pairs exceeded the concordance of the best-performing technical replicate, demonstrating that the collection harbors genetically indistinguishable or extremely closely related accessions, rather than indicating limited marker resolution.

### Functional annotation of SNPs in key metabolic pathways

**Figure 8:**
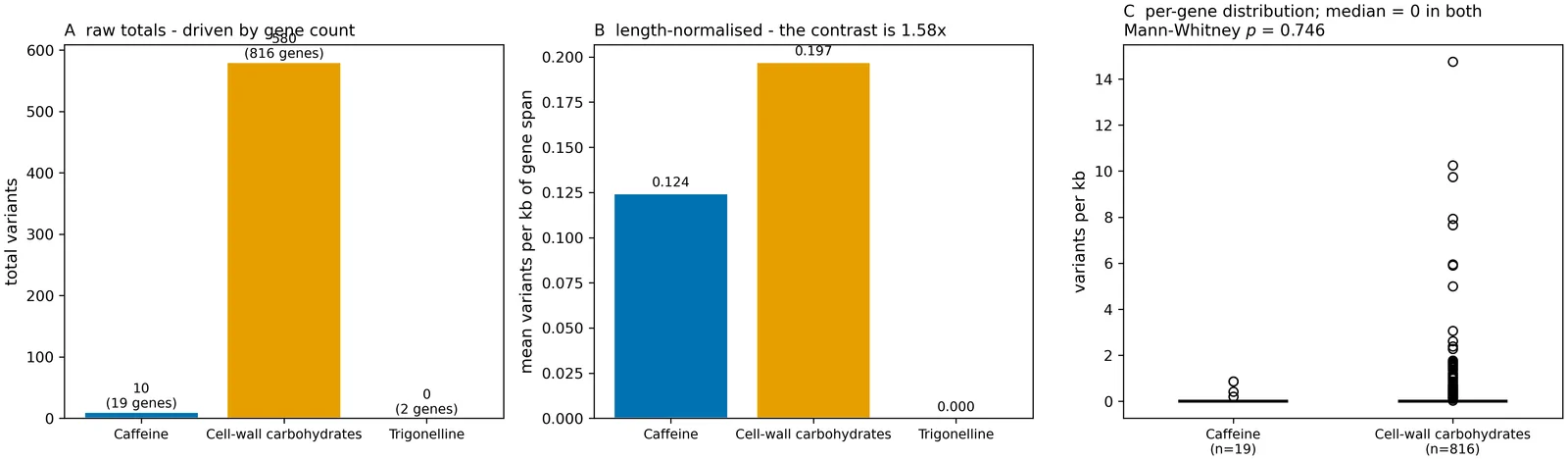
Variant distribution in candidate metabolic pathways. **(A)** Total number of variants identified in genes associated with caffeine biosynthesis and cell-wall carbohydrate metabolism. **(B)** Variant density normalized by gene length (variants kb^−1^). **(C)** Distribution of normalized variant density per gene in the two pathway categories.

Genes involved in caffeine biosynthesis, cell-wall carbohydrate metabolism, and trigonelline biosynthesis were screened for sequence variants (**Figure 8**; **Supplementary Table 8**). A total of 10 variants were identified across 19 caffeine biosynthesis genes and 580 variants across 816 cell-wall carbohydrate metabolism genes. However, these differences largely reflected the contrasting numbers of genes in each category. After normalization by gene length, variant densities were similar (0.124 and 0.197 variants kb^−1^, respectively), and the difference was not statistically significant (Mann–Whitney *U* test, raw *p* = 0.675; normalized *p* = 0.746; Benjamini–Hochberg adjusted *p* = 0.746 for both comparisons). Median variant counts per gene were zero in both categories. No variants were detected in the two trigonelline biosynthesis genes, precluding statistical comparison.

## Discussion

The conservation and genetic characterization of Saudi coffee accessions are essential for sustaining production under arid and semi-arid environments (Alhudaib and Ismail, 2024). Given the narrow genetic base and predominantly self-pollinating nature of *Coffea arabica*, maintaining and expanding genetic diversity is critical for long-term resilience and breeding progress (Khemira et al., 2023a). In this context, genome-wide approaches provide a powerful framework for uncovering hidden diversity and guiding conservation strategies.

The most consequential finding of this study is the identification of extensive subgenome cross-mapping artefacts, which produced artificially high heterozygosity across the *C. arabica* genome. This likely reflects the high sequence similarity between the recently diverged homoeologous subgenomes and should be considered in future resequencing studies (Mekbib et al., 2022).

Correcting for the cross-mapping artefact reversed the apparent excess of observed over expected heterozygosity, yielding positive FIS values consistent with the predominantly self-pollinating reproductive system of *C. arabica*. These findings indicate that the previously observed excess heterozygosity was primarily a technical artefact rather than evidence of residual outcrossing, historical hybridization, or gene flow (Cahyono et al., 2024). Because the filtering procedure directly targets highly heterozygous loci, the corrected values should be regarded as indicative of the direction and magnitude of the artefact rather than as unbiased estimates of inbreeding (Jiang et al., 2025). Collectively, these findings suggest that Saudi coffee germplasm harbors greater genetic complexity than previously assumed, which is advantageous for breeding but requires careful management (Niyoyankunze et al., 2023).

Population structure was largely unaffected by correction of the cross-mapping artefact. ADMIXTURE and PCA produced highly consistent patterns before and after filtering, with seven ancestry groups showing no clear correspondence to the three collection regions. Together with the low regional differentiation detected by AMOVA and the interspersion of accessions in the phylogeny, these findings indicate that the genetic structure reflects relationships among *ex situ* germplasm shaped by propagation and exchange rather than geographic subdivision. The concordance among ADMIXTURE, PCA, and phylogenetic analyses indicates that the collection is characterized by structured but continuous genetic variation rather than discrete regional groups, consistent with previous studies of *C. arabica* germplasm (Zhang et al., 2024; Al-Ghamedi et al., 2023). From a germplasm management and breeding perspective, the coexistence of admixed accessions and genetically differentiated groups provides a valuable resource for selecting diverse parental lines and broadening the genetic base of breeding populations (Liu et al., 2020; Verleysen et al., 2024).

Standard IBD- and KING-based relatedness estimators were unsuitable for these data because subgenome cross-mapping violated their underlying assumptions, highlighting the need for homoeolog-aware read mapping and variant-calling strategies to improve relatedness analyses in allotetraploid *C. Arabica* (Salojärvi et al., 2023).

The candidate 150-SNP fingerprinting panel showed high informativeness, with a mean Botstein PIC of 0.374, representing 99.6% of the theoretical maximum for biallelic markers. Its principal limitation lies not in marker performance but in the composition of the germplasm collection. Several non-replicate accession pairs exhibited very high genotype concordance, including one pair that was indistinguishable across all successfully genotyped loci, indicating genuine redundancy within the collection. The panel is therefore well suited for accession identification, germplasm management, and conversion into cost-effective genotyping platforms such as KASP assays (Mannino et al., 2023; Jiang et al., 2025). However, independent validation using genetically diverse and unrelated *C. arabica* accessions is required before routine deployment. The redundancy detected by the panel provides valuable information for rationalizing gene-bank collections and improving the efficiency of germplasm conservation.

Finally, the pathway annotation highlights the importance of accounting for gene category size when comparing variant abundance. Raw SNP counts were higher in cell-wall carbohydrate metabolism genes than in caffeine biosynthesis genes, but this difference was largely explained by the much larger number of genes in the former category. After normalization by gene length, the difference was modest and not statistically significant; therefore, we do not infer differential functional constraint from these data. Nevertheless, caffeine biosynthesis and cell-wall carbohydrate metabolism remain important determinants of coffee quality and adaptation (Raharimalala et al., 2021; Tavares Flores et al., 2026), and future studies integrating genome-wide variation with transcriptomic and phenotypic data will be required to assess the functional significance of variants in these pathways.

Several limitations should be acknowledged. First, the sequencing depth (~11.5×) is modest for an allotetraploid genome and may have reduced the accuracy of genotype calling in highly similar homoeologous regions. Second, because the artefact filter was based on observed heterozygosity, the corrected diversity statistics should be interpreted as indicative rather than unbiased estimates. Third, variant density was normalized by gene span rather than coding sequence length. Finally, no outgroup was included, and the phylogeny is therefore presented as unrooted.

## Conclusion

Whole-genome resequencing of the Saudi *Coffea arabica* germplasm collection revealed seven robust ancestry groups that reflect relationships among *ex situ* accessions rather than geographic origin, with no significant genetic differentiation among the three collection regions. We identified and corrected a subgenome cross-mapping artefact that artificially inflated genome-wide heterozygosity and obscured the predominantly self-pollinating nature of *C. arabica*. We also developed a high-quality candidate 150-SNP fingerprinting panel that provides a valuable resource for accession identification and germplasm management, while highlighting the need for independent validation before routine application. Overall, this study provides a robust genomic framework for the conservation and management of Saudi coffee genetic resources and demonstrates the importance of subgenome-aware analytical approaches for whole-genome studies of allotetraploid *C. arabica*.

## Data Availability

The datasets presented in this study can be found in online repositories. The names of the repository/repositories and accession number(s) can be found in the article/**Supplementary Material**.

## Supplementary material

Supplementary Figure 1 Distribution of per-site observed heterozygosity in dataset A, showing a bimodal pattern with a distinct density trough used to define the heterozygosity filtering threshold.

Supplementary Figure 2 Allele balance and read depth at sites with high and normal observed heterozygosity.

Supplementary Figure 3 Machine-readable QR code genotype fingerprints for the 78 Coffea arabica accessions, generated from the 150-marker candidate panel. Shown as a composite plate for reference, the individual full-resolution codes are provided as supplementary data.

Supplementary Figure 4 SNP variation in coffee accessions. SNP transitions and transversions distribution with a global Ts/Tv ratio of 1.91.

Supplementary Figure 5 Quality-control filtering cascade. Variant-level and sample-level filtering are shown as separate tracks, since the two are produced by different operations.

Supplementary Figure 6 Equal-angle unrooted representation of the maximum-likelihood phylogeny shown in **Figure 3**, illustrating the tree topology without a rooting assumption.

Supplementary Figure 7 Principal component analysis based on the unfiltered Dataset A, shown for comparison with the heterozygosity-filtered analysis in **Figure 4**.

Supplementary Figure 8 Degeneracy of the PI_HAT and KING kinship estimators before and after cross-mapping artefact removal.

Supplementary Figure 9 Identity-by-state similarity among all 3, 655 pairwise comparisons. **(A)** Stability of the measure before and after removal of the cross-mapping artifact. **(B)** Distribution for different accessions, with the eight technical replicate pairs marked. This figure combines the two separate identity-by-state figures of the previous version, which presented overlapping information.

Supplementary Table 1 Summary of the 78 Saudi coffee accessions with corresponding place of collection.

Supplementary Table 2 Raw sequencing read quality statistics for the 86 libraries.

Supplementary Table 3 Post-filtering sequencing read quality statistics for the 86 libraries.

Supplementary Table 4 Correspondence between the chromosome nomenclature used in this study and the native subgenome-specific chromosome names of the *Coffea arabica* reference genome (Cara_r6).

Supplementary Table 5 Mapping and genome coverage statistics for the 86 sequencing libraries.

Supplementary Table 6 Marker statistics for the 150-marker candidate fingerprinting panel, giving minor allele frequency, expected heterozygosity (2pq), Botstein PIC, and observed heterozygosity for each marker.

Supplementary Table 7 Genotype calls for the 150-marker candidate fingerprinting panel across 78 *Coffea arabica* accessions. Markers are identified by their genomic coordinates (chromosome:position); the anonymized marker labels (SNP_1–SNP_150) used in the submitted version have been replaced with their corresponding genomic positions.

Supplementary Table 8 Variants identified in genes associated with caffeine biosynthesis, cell-wall carbohydrate metabolism, and trigonelline biosynthesis, including gene length-normalized variant densities.

Supplementary Table 9 Traceability of the eight technical replicate libraries (C27, C30, C32, C33, C38, C39, C62, and C93), linking each to its parent accession in **Supplementary Table 1** through the shared gene bank code, with the observed identity-by-state similarity, its rank among all 3, 655 pairwise comparisons, and the panel concordance for that pair.

Supplementary Table 10 Validation of the per-accession QR code fingerprints, reporting payload length, QR version, and the result of decoding each rendered code back to its input payload.
